# Systematic design and evaluation of aptamers for VEGF and PlGF biomarkers of Preeclampsia

**DOI:** 10.1186/s12896-024-00891-0

**Published:** 2024-09-27

**Authors:** Samavath Mallawarachchi, Rümeysa E. Cebecioglu, Majed Althumayri, Levent Beker, Sandun Fernando, Hatice Ceylan Koydemir

**Affiliations:** 1 Department of Biological and Agricultural Engineering, Texas A&M University, College Station, TX 77843 USA; 2 Department of Biomedical Sciences and Engineering, Koç University, Istanbul, 34450 Turkey; 3 Department of Mechanical Engineering, Koç University, Istanbul, 34450 Turkey; 4 Department of Biomedical Engineering, Texas A&M University, College Station, TX 77843 USA; 5 Center for Remote Health Technologies and Systems, Texas A&M Engineering Experiment Station, College Station, TX 77843 USA; 6 Medical Laboratory Techniques, Health Services of Vocational School, Kent University, Istanbul, 34333 Turkey

**Keywords:** Preeclampsia, VEGF, PlGF, Aptamer, Diagnostics, Aptasensor, Bio-layer interferometry, Molecular docking, Aptamer design, Biomarker

## Abstract

Preeclampsia is a potentially life-threatening condition for both mother and baby, characterized by hypertension and potential organ damage. Early diagnosis is crucial to mitigate its adverse health effects. Traditional diagnostic methods, which focus on late-manifesting symptoms like hypertension and proteinuria, underscore the need for molecular diagnostic approaches for timely detection. This study successfully designs and evaluates novel aptamers with high specificity and affinity for Vascular Endothelial Growth Factor (VEGF) and Placental Growth Factor (PlGF), biomarkers closely associated with preeclampsia. Using molecular docking, molecular dynamics simulations, and BioLayer Interferometry (BLI), we identified aptamers that demonstrated strong binding affinities, comparable or superior to traditional antibodies. Our findings suggest that these aptamers have the potential to be integrated into cost-effective, point-of-care diagnostic tools, significantly improving early detection and intervention strategies for preeclampsia. The robust performance of these aptamers marks a pivotal step toward the development of more reliable and accessible diagnostic solutions, with implications for better maternal and fetal health outcomes.

**Supplementary Information:**

The online version contains supplementary material available at 10.1186/s12896-024-00891-0.

## Background

Preeclampsia is a complex pregnancy disorder affecting maternal and neonatal health, potentially life-threatening if not diagnosed early. It is marked by hypertension and potential organ damage, especially to the liver and kidneys, and is tied to diverse pathophysiological processes [1–4]. Oral and IV medications to lower blood pressure can be administered to the women to treat the disease until the baby is mature enough to be delivered. In the U.S., it affects about 4% of pregnancies [5], while in parts of Europe, rates approach 10% [6, 7], causing more than 50,000 maternal deaths and half a million fetal deaths worldwide [8].

Current diagnostic criteria, primarily hypertension, and proteinuria, often manifest late (usually around 20 weeks of pregnancy), narrowing intervention opportunities and increasing risks of prematurity and intrauterine death of the fetus [9]. Overlaps with other hypertensive pregnancy disorders further complicate the diagnosis of preeclampsia [10, 11]. Preeclampsia has no specific symptoms and is detected indirectly through routine tests before delivery and symptom evaluation [12], such as problems with vision, vomiting, sudden swelling of hands or face, and severe headache, making the clinical diagnosis challenging. This underscores the urgent need for enhanced molecular diagnostic approaches for early detection of preeclampsia and to provide appropriate treatment to save lives [13, 14].

Several molecular markers have been investigated to aid in accurately diagnosing preeclampsia [15–17]. Among these biomarkers, Vascular Endothelial Growth Factor (VEGF) and Placental Growth Factor (PlGF) proteins are prominent due to their roles in the angiogenic imbalance characteristic of preeclampsia [18]. In typical pregnancies, VEGF and PlGF regulate placental vascular development, ensuring mother-fetus nutrient exchange [19]. However, traditional diagnostic methods for preeclampsia rely heavily on late-manifesting symptoms such as hypertension and proteinuria, which limit early intervention opportunities [9]. This necessitates the development of molecular diagnostic tools that can detect preeclampsia earlier in pregnancy, potentially improving maternal and fetal outcomes [13, 14].

VEGF regulates blood vessel development, balancing, and stabilization by signaling with other angiogenic factors during normal pregnancy [20]. It also protects endothelial cell functions of the brain and glomeruli, which are severely affected organs in the case of preeclampsia. In preeclampsia, excessive anti-angiogenic factors secreted from the placenta decrease VEGF signalling, impairing endothelial cell functionality [21]. The prenatal serum VEGF level in pregnant women with preeclampsia was reported at 51.7 ng/mL, compared to 13.9 ng/mL in the control group [22]. PlGF, a member of the VEGF protein family, sharing about 53% similarity with VEGF [23], is critical for placental angiogenesis and early trophoblast growth. Primarily found in the placenta, its blood concentration rises during pregnancy to support placental blood vessel growth, peaking between 12 and 30 weeks, and then dropping, ranging from 141 pg/mL to 23 pg/mL [24]. Women with preeclampsia exhibit decreased PlGF levels in blood and urine [25], and PlGF concentration fluctuations can diagnose preeclampsia with about 90% accuracy.

Regulating angiogenesis involves the relationship between VEGF and PlGF. PlGF competitively binds to the VEGFR-1 receptor to increase VEGF activity, enabling stronger VEGF binding to the VEGFR-2 receptor. However, PlGF and VEGF can form a heterodimer with pro-angiogenic or anti-angiogenic effects. Evaluating VEGF and PlGF together is essential for diagnosing preeclampsia, given this process’s role in its development [26].

Various analytical techniques, such as fluorescence spectrometry and enzyme-linked immunosorbent assay (ELISA), have been investigated to detect VEGF and PlGF [27–30]. These techniques often require antibodies for specific biomarker detection. Unlike traditional antibodies, aptamers—single-stranded RNA, DNA, or peptide molecules with distinct three-dimensional structures—offer advantages like room temperature stability, protease resistance, and the ability to undergo multiple denaturation and renaturation cycles, making them suitable for prolonged use and storage [31–33]. Additionally, while antibody affinity can be epitope-dependent, aptamers consistently show high specificity, making them promising recognition elements for point-of-care sensing technologies [34–36].

In this study, we designed and evaluated the specific aptamer sequences with a high affinity for VEGF and PlGF proteins, using molecular docking followed by experimental validation with BioLayer Interferometry. Through this study, we provide a theoretical basis for the early diagnosis of preeclampsia using biosensors. By investigating the binding dynamics of VEGF and PlGF aptamers, we aim to establish foundational insights that could inform the development of sensitive and specific diagnostic tools. Our expectation is that these preliminary findings will contribute to the ongoing efforts to enhance early detection and intervention strategies for preeclampsia. However, we recognize that further optimization and validation are necessary to confirm these aptamers’ efficacy and reliability in clinical settings.

## Results and discussion

### Aptamer design

As the initial step in the aptamer design process, nucleotides with terminals modified to emulate their behavior in bound form were docked on the receptors. Adenosine monophosphate (AMP), guanosine monophosphate (GMP), and cytidine monophosphate (CMP) showed Glide docking scores between − 4 and − 5 kcal/mol, and Glide energies lower than − 25 kcal/mol with both receptors. Other aptamer design studies in the literature indicate docking scores around − 7 and − 8 kcal/mol as strong binding, suggesting that the nucleotides display moderately strong binding to the receptors [37, 38]. CMP demonstrated the strongest binding to both receptors, and uridine monophosphate (UMP) showed the weakest binding. Statistical analysis of Glide energies (Tables S1 and S2) revealed that the binding of UMP was significantly weaker than other nucleotides for both PlGF and VEGF. Docking scores for the top binding configurations of each nucleotide on PlGF and VEGF are given in Table 1.

**Table 1:** Docking scores and Glide energies of the nucleotides on PlGF and VEGF. All values are expressed as mean ± standard deviation based on the three strongest binding conformations

Nucleotide	With PlGF		With VEGF	
	Docking score (kcal/mol)	Glide Energy (kcal/mol)	Docking score (kcal/mol)	Glide energy (kcal/mol)
CMP	-5.277 ± 0.167	-35.862 ± 0.840	-4.100 ± 0.614	-25.108 ± 0.710
AMP	-5.066 ± 0.101	-35.911 ± 0.162	-3.417 ± 0.097	-25.743 ± 1.358
GMP	-4.653 ± 0.535	-35.153 ± 2.624	-3.662 ± 0.525	-25.145 ± 1.883
UMP	-4.446 ± 0.156	-30.130 ± 0.559	-3.382 ± 0.128	-22.573 ± 0.425

**Fig. 1:**
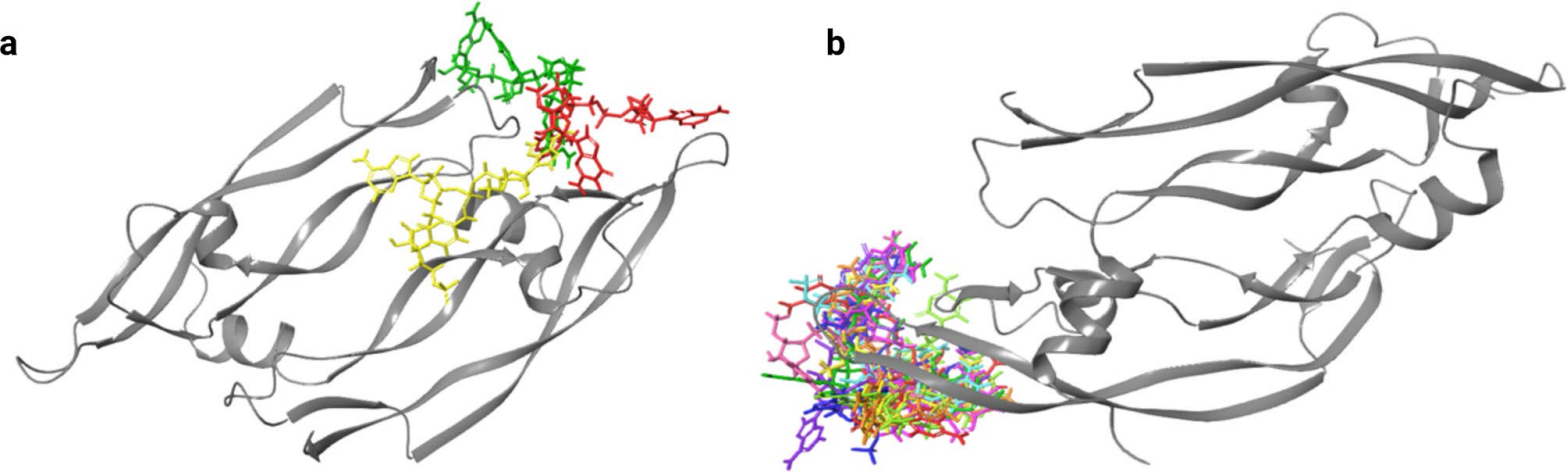
(**a**) The strongest binding trimer in each cluster on PlGF: AGG (red), CAA (yellow) and GCA (green); and (**b**) Binding conformations of strongest binding trimers on VEGF: AGG (red), AAA (orange), CGG (yellow), CGA (light green), GAA (green), CCG (light blue), GGA (blue), GAC (purple), GGG (magenta), CAC (pink)

To identify the strongest binding nucleotide combinations, dimers and trimers consisting of CMP, AMP, and GMP were docked on the receptors. In general, trimers showed stronger binding than dimers to both receptors, which can be attributed to a higher number of interactions. On PlGF, it could be observed that the dimers and trimers are bound to three different locations within the grid. In comparison, no separate clusters could be observed on VEGF, and all trimers bound to approximately the same region. Docking results for the strongest binding trimers on PlGF and VEGF are given in Table 2, and the binding conformations of the strongest binding trimers on the receptors are visualized in Fig. 1.

According to Table 2, the GGG was the strongest binding trimer on cluster 1 and overall PlGF protein, with an average docking score of -7.113 kcal/mol and an average Glide docking energy of -66.900 kcal/mol, which is indicative of very tight binding. ACA was the strongest binding trimer in cluster 3 (docking score of -6.889 kcal/mol) and showed slightly weaker binding than GGG, while GCA, the strongest binding trimer in cluster 2 showed moderately strong binding to PlGF (docking score of -5.050 kcal/mol). According to Fig. 1a; Table 2, some overlapping could be observed between clusters, with some residues, including TYR33 and ASP42, forming interactions with trimers binding to different clusters.

**Table 2:** Docking scores, Glide energies, and H-bond interactions of the strongest binding trimers on PlGF and VEGF. All values are expressed as mean ± standard deviation based on the three strongest binding conformations

Aptamer	Docking score (kcal/mol)	Glide Energy (kcal/mol)	H bond interactions	Cluster
**For PlGF**				
AGG	-6.734 ± 1.231	-63.931 ± 7.338	VAL51, HIS53, SER56, ASP71, GLU72, GLY94	1
GGG	-7.113 ± 0.876	-66.900 ± 4.451	VAL51, GLU52, PHE55, SER56, SER58, GLU72	1
CAA	-5.613 ± 1.944	-57.960 ± 11.160	ARG39, LEU40, ASP42, SER58, GLY67	3
GCG	-6.991 ± 1.020	-66.597 ± 3.083	TYR33, VAL51, GLU52, ASP71, GLU72	1
ACA	-6.889 ± 0.238	-69.595 ± 1.999	ARG38, SER58, GLY67, LEU74	3
GAG	-5.313 ± 0.920	-65.236 ± 5.829	ARG39, ASP42, GLY67, ASP71, GLU72	3
GCA	-5.050 ± 1.275	-51.232 ± 4.580	TYR33, PHR55, CYS69, GLY70, GLU72, CYS112	2
GCC	-4.976 ± 0.247	-55.050 ± 3.267	GLY30, TYE33, VAL108	2
**For VEGF**				
AGG	-7.159 ± 0.214	-56.639 ± 2.186	GLU44, LYS84, HIS86, GLN87	Not applicable
AAA	-6.626 ± 1.094	-56.234 ± 9.408	GLU44, ARG82, LYS84, GLN87	Not applicable
CGG	-6.217 ± 0.907	-51.246 ± 8.171	GLU42, GLU44, ARG82, LYS84, GLN87	Not applicable
CGA	-6.883 ± 0.196	-57.602 ± 2.794	ASP41, GLU44, TYR45, LYS84, GLN87	Not applicable
GAA	-7.016 ± 0.025	-59.915 ± 0.999	GLU42, GLU44, LYS84, GLN87	Not applicable
CCG	-6.181 ± 0.747	-47.148 ± 2.715	GLU42, GLU44, LYS84, HIS86, GLN87	Not applicable
GGA	-6.261 ± 0.610	-53.213 ± 3.850	ASP41, GLU44, LYS84, GLN87	Not applicable
GAC	-6.373 ± 0.326	-52.159 ± 2.615	GLU42, GLU44, ARG82, LYS84, PRO85, GLN87	Not applicable
GGG	-6.572 ± 0.099	-51.142 ± 0.420	ASP41, GLU42, GLU44, LYS84, GLN87	Not applicable
CAC	-6.103 ± 0.455	-54.774 ± 0.997	LYS84, PRO85, GLN87	Not applicable

As illustrated in Fig. 1b, no clusters could be observed on VEGF, and all the short aptamers bound to approximately the same region. It also agrees with the analysis of the interactions in Table 2, which shows that residues such as GLU42, GLU44, GLY84, and GLN87 in VEGF have formed H-bond interactions with the majority of the aptamers. Among the trimers targeting VEGF, AGG showed the strongest binding, with an average docking score of -7.159 kcal/mol and a Glide energy of -56.639 kcal/mol. While the docking scores and Glide energies of the trimers targeting VEGF were slightly higher than those of PlGF, most trimers showed strong binding to VEGF, with docking scores comparable to strong binding aptamers in other studies [37, 38].

Four long aptamers with a potentially strong affinity towards PlGF and six aptamers with a potentially strong affinity towards VEGF were designed by joining the trimers using different combinations, and the binding of those aptamers to the receptors was evaluated based on vina docking scores and MM-GBSA energies. Sequences and binding information for the long aptamers on PlGF and VEGF are given in Table 3.

**Table 3:** Sequences of the aptamers designed for PlGF and VEGF, and MM-GBSA energies and H-bond interactions of the strongest binding configurations of those aptamers. All values are expressed as mean ± standard deviation based on the three strongest binding conformations

Aptamer	Sequence	Vina docking score	MM-GBSA Energy (kcal/mol)	MM-GBSA Energy_No Strain (kcal/mol)	H-bond interactions
**For PlGF**					
PlGF-Apt1	AACAGGCAA	7.90 ± 0.61	-4.16 ± 8.42	-44.07 ± 6.57	ARG35, GLU38
PlGF-Apt2	ACAGGCACA	-3.53 ± 1.15	-13.21 ± 25.98	-43.74 ± 9.76	GLU38, SER45, CYS76, SER105
PlGF-Apt3	AGAGAACGCAAGAGA	98.85 ± 16.90	-18.85 ± 7.81	-58.99 ± 12.42	GLN26, ARG35, GLU38, ARG39, THR66, CYS76, SER105
PlGF-Apt4	CGAAGAGACGCAGAGAAGC	Did not bind	10.92 ± 24.89	-44.96 ± 18.11	GLN26, GLU38, SER45, ARG64, CYS76, HIE107
**For VEGF**					
VEGF-Apt 1	CGAAGGGAA	-1.00 ± 0.26	6.32 ± 38.16	-28.60 ± 32.98	GLN79, HIS86, GLN87, GLY88, GLN89
VEGF-Apt 2	CGAAAACAC	-2.00 ± 0.35	1.62 ± 24.61	-39.75 ± 17.12	HIS86, GLN87, GLN89, GLU93
VEGF-Apt 3	GAAAGGCGA	-2.73 ± 0.21	17.20 ± 6.06	-12.62 ± 4.73	HIS86, GLN87, GLN89, HIS90, GLU93
VEGF-Apt 4	GAAAGGCAC	-1.90 ± 0.00	1.12 ± 10.38	-21.88 ± 5.12	TYR45, GLN79, LYS84, HIS86, GLN87, GLY88, GLN89, HIS90
VEGF-Apt 5	AAAAGGCGG	-1.80 ± 0.26	2.36 ± 8.26	-10.95 ± 8.50	LYS48, HIS86, GLN87, GLN89, HIS90, ILE91
VEGF-Apt 6	CGACGAAGGGAACAC	-0.77 ± 0.21	14.75 ± 5.52	-14.01 ± 7.29	LYS48
V7t1 (control)	TGTGGGGGTGGACGGGCCGGGTAGA	Did not bind	17.97 ± 18.44	-6.86 ± 6.48	LYS48, HIS86, GLY88

Among the long aptamers targeting PlGF, PlGF-Apt3 showed the strongest binding, with an average MM-GBSA binding energy of -18.85 kcal/mol, with PlGF-Apt2 also showing strong binding. The strong binding of PlGF-Apt2 was also evident in Autodock vina results, with an average docking score of -3.53 kcal/mol. In comparison, other aptamers showed much weaker binding in Autodock vina, with positive docking scores. Analysis of interactions showed that all aptamers except PlGF-Apt1 formed more than three hydrogen bonds with PlGF. Among the residues in PlGF, ARG35, GLU38, SER45, CYS76, and SER105 could be identified as critical residues which formed H-bonds with multiple aptamers. Based on docking scores, MM-GBSA energies and the number of interactions, PlGF-Apt2, PlGF-Apt3, and PlGF-Apt 4 were screened as candidates for experimental validation.

It can be observed in Table 3 that the aptamers targeting VEGF showed generally weaker affinity towards the receptor compared to the aptamers targeting PlGF, as observed by positive mean MM-GBSA energies and high standard deviations. However, the aptamers showed a negative MM-GBSA energies when ligand strain was not considered, suggesting that they have a chance of binding to VEGF if they can be properly aligned to the binding configuration. Additionally, it could also be observed that all these aptamers showed lower MM-GBSA energies than v7t1, which has been reported to have a high affinity to VEGF in literature [39]. Among the long aptamers targeting VEGF, VEGF-Apt1, and VEGF-Apt2 demonstrated the strongest binding conformations to VEGF, with significantly negative no-strain MM-GBSA energies. Among all aptamers, VEGF-Apt3 showed the smallest standard deviation of MM-GBSA energy, suggesting it may demonstrate a higher specificity towards VEGF. According to Autodock vina results, VEGF-Apt3 showed the strongest binding, followed by VEGF_Apt2 and VEGF_Apt4. All aptamers except VEGF-Apt6 formed multiple H-bonds with VEGF, suggesting that shorter aptamers are more likely to bind strongly to VEGF. Based on the vina docking scores and MM-GBSA binding energies, VEGF-Apt1, VEGF-Apt2, and VEGF-Apt3 were selected for experimental validation.

### Molecular dynamics simulations

**Fig. 2:**
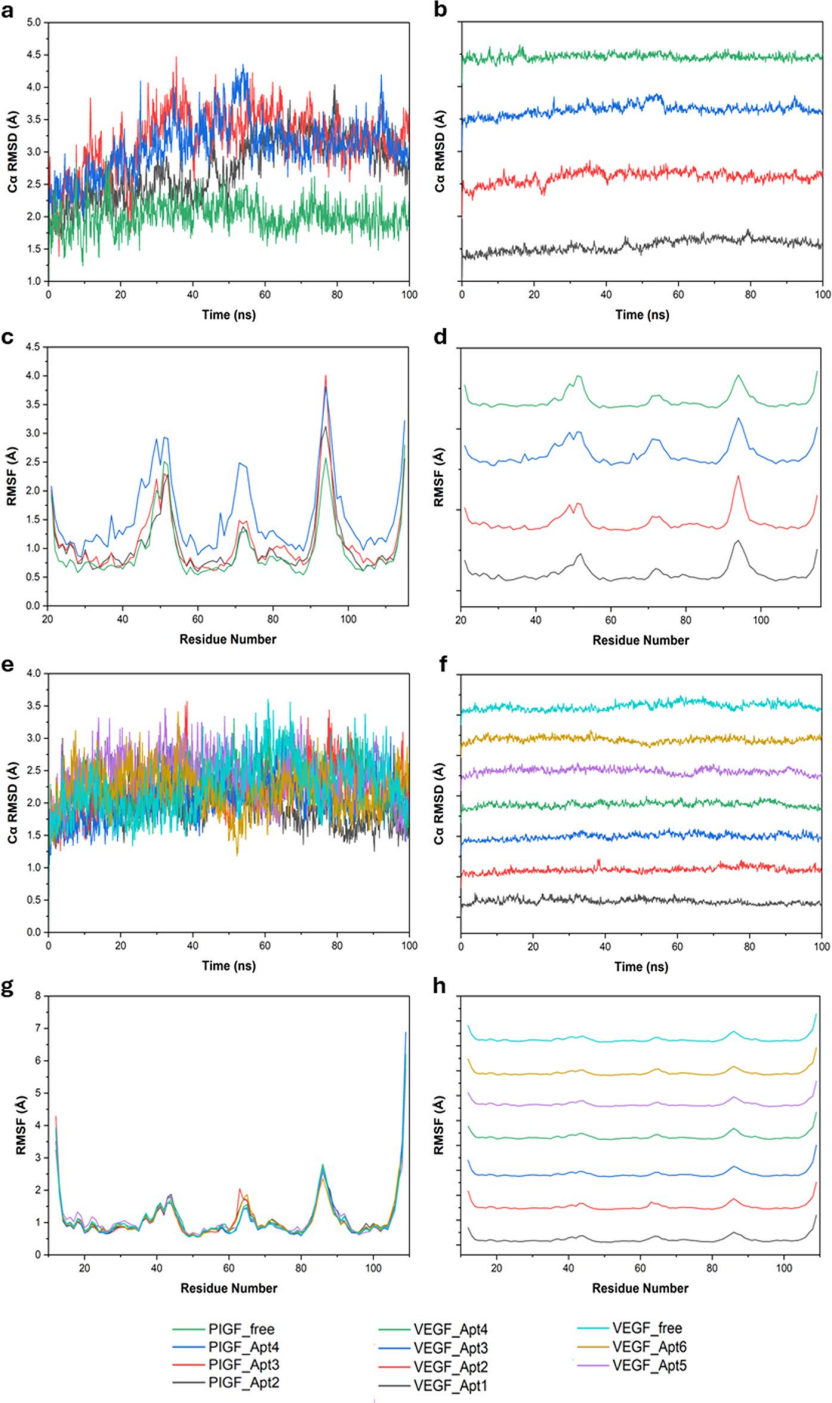
(**a**) RMSD diagram and (**b**) stacked line RMSD plot of PlGF complexed with aptamers; (**c**) RMSF diagram and (**d**) stacked line RMSF plot of PlGF complexed with aptamers; (**e**) RMSD diagram and (**f**) stacked line RMSD plot of VEGF complexed with aptamers; (**g**) RMSF diagram and **d**) stacked line RMSF plot of VEGF complexed with aptamers

The stability of aptamer binding to PlGF and VEGF was analyzed via molecular dynamics simulations. Root Mean Square Deviation (RMSD) and Root Mean Square Fluctuation (RMSF) diagrams for the aptamer-receptor complexes are presented in Fig. 2.

As shown in Fig. 2a and b, the RMSD of PlGF has increased when complexed with the aptamers, with all three PlGF-aptamer complexes exhibiting higher RMSD than the free protein towards the end of the simulation, which suggests increased flexibility upon binding. Simulations for all three aptamers stabilized after 60 ns, at an RMSD around 3 Å, which indicates stable binding for an aptamer or a large biomolecule [40–42]. According to RMSF analysis (Fig. 2c and d), PlGFApt2 and PlGF_Apt3 showed RMSF values similar to the free protein, while PlGF_Apt4 showed a significantly higher RMSF. No noticeable shifts in RMSF peaks were observed for any of the aptamers, indicating that the flexibility of the receptor remains conserved upon aptamer binding [43]. Analysis of trajectories (trajectory videos included under supplementary data) revealed that one terminus of the aptamers remained stably bound to PlGF, while the other terminus remained free, making these aptamers promising candidates for sensing purposes.

Aptamers targeting VEGF showed more stable binding than those targeting PlGF, as depicted by the RMSD and RMSF diagrams (Fig. 2e and h), which show no significant shifts of RMSD or RMSF upon aptamer binding. RMSD plots for all aptamers except VEGF_Apt5 and VEGF_Apt6, have stabilized after 60 ns at around 2 Å, indicating stable binding [40–42]. These results agree with the trajectory videos, which demonstrate that all aptamers except those two achieve stability towards the end of the simulation. Therefore, based on MD simulations VEGF_Apt1, VEGF_Apt2, VEGF_Apt3 and VEGF_Apt4 can be identified as stable binding aptamers.

### Bio-layer interferometry

The bindings of the aptamers on PlGF and VEGF were evaluated based on association rate (Ka), dissociation rate (Kd), and affinity constant (KD). Binding kinetics parameters for the aptamers on PlGF are given in Table 4, and the BLI graphs for aptamer binding on PlGF are given in Fig. S1.

According to Table 4, PlGF-Apt3 clearly shows stronger binding than the other two aptamers, with a nano-molar range affinity (smaller affinity constants mean stronger binding). The association rate of PlGF-Apt3 is slightly lower than the other two aptamers, but the dissociation rate of PlGF-Apt3 is much smaller. This means that once bound to PlGF, PlGF-Apt3 dissociates very slowly, which suggests tight binding. This can also be observed in Fig. S1b, which shows nearly horizontal dissociation curves. In comparison, PlGF-Apt2 and PlGF-Apt4 have shown high dissociation rates, which is also depicted in the fast dissociation curves for those two aptamers (Fig. S1a and S1c). This suggests that the binding of PlGF-Apt2 and PlGF-Apt4 is weaker than PlGF-Apt3. Statistical analysis of BLI results (Tables S3 and S4) confirmed that PlGF-Apt3 showed significantly smaller KD and Kd values than the other two aptamers (*p* < 0.0001). Thus, BLI results suggest PlGF-Apt3 as a strong binding aptamer to PlGF protein.

**Table 4:** Association rates, dissociation rates, and affinity constants of the aptamers of PLGF and VEGF based on three replicates. All values are expressed as mean ± standard deviation

Aptamer	Affinity constant (K_D_) (M)	Association rate (K_a_) (1/M_s_)	Dissociation rate (K_d_) (1/s)
PlGF-Apt2	(1.774 ± 1.763) × 10^− 5^	(1.772 ± 0.934) × 10^4^	(2.049 ± 0.780) × 10^− 1^
PlGF-Apt3	(2.983 ± 1.815) ×10^− 10^	(2.746 ± 2.329) × 10^3^	(5.548 ± 1.954) × 10^− 7^
PlGF-Apt4	(4.969 ± 4.840) ×10^− 5^	(3.169 ± 2.377) × 10^3^	(2.150 ± 2.715) × 10^− 1^
VEGF-Apt1	(3.203 ± 5.535) × 10^− 4^	(1.100 ± 0.941) × 10^3^	(5.647 ± 7.748) × 10^− 3^
VEGF-Apt2	(4.671 ± 6.860) ×10^− 10^	(0.905 ± 1.208) × 10^4^	(4.708 ± 0.303) × 10^− 7^
VEGF-Apt3	(6.804 ± 4.655) ×10^− 9^	(9.164 ± 7.377) × 10^1^	(4.362 ± 0.788) × 10^− 7^

Binding kinetic parameters for the aptamers on VEGF and the association and dissociation curves for the aptamer binding on VEGF are given in Table 4 and Fig. S2, respectively. According to Table 4, VEGF-Apt1 has a high affinity constant and a dissociation rate, which suggests that it does not stay bound tightly to VEGF compared to the other two aptamers. This can also be observed in Fig. S2a. The difference between affinity constants and dissociation rates for VEGF-Apt1 and the other two aptamers was also evident in the student’s t-test (Tables S5 and S6). Among the other two aptamers, VEGF-Apt2 has the smallest average affinity constant, suggesting it binds strongly to VEGF. However, Table 4 shows that VEGF-Apt2 displays unusually high standard deviation in both affinity constant and dissociation rate. In addition, in Fig. S2b, some degree of association occurs even during the dissociation rate. Both these observations suggest significant non-specific binding of VEGF-Apt2. In comparison, VEGF-Apt3 has a higher affinity constant but a lower standard deviation and also shows stable binding in the BLI curve (Fig. S2c). The low dissociation rate of VEGF-Apt3 suggests that while its binding is slower than VEGF-Apt2, it remains tightly bound to VEGF. Considering kinetic parameters and BLI curves, VEGF-Apt3 seems to be the most suitable candidate, while VEGF-Apt2 also shows strong binding. Overall, both these aptamers showed nanomolar level binding, which is comparable to some of the VEGF binding aptamers reported in the literature, as given in Table S7.

## Materials and methods

### Aptamer sequences and his-tagged proteins

Aptamers specific to PlGF and VEGF were purchased from Aptagen (Jacobus, PA, USA), with each sequence at a concentration of 0.5 µmole RNA and standard desalting. The VEGF aptamers were Aptamer 1 {5’-CGAAGGGAA-3’}, Aptamer 2 {5’-CGAAAACAC-3’}, and Aptamer 3 {5’-GAAAGGCGA-3’}. For PlGF, the sequences were Aptamer 2 {5’-ACAGGCACA-3’}, Aptamer 3 {5’-AGAGAACGCAAGAGA-3’}, and Aptamer 4 {5’-CGAAGAGACGCAGAGAAGC-3’}. Additionally, specific his-tagged proteins for PlGF (Human PlGF / PGF (19–149) Protein, His Tag, product no. PGF-H52H5) and VEGF (Human VEGFR2 / KDR Protein, His Tag, KDR-H5227) were obtained from Acrobiosystems (Newark, DE, USA). All aptamers and proteins were suspended in buffer solutions, aliquoted and stored at -80 °C to maximize shelf life, following the manufacturer’s instructions to maintain their activity throughout the experiments.

### Protein and aptamer structures

Structures of PlGF and VEGF were obtained from the Research Collaboratory for Structural Bioinformatics RCSB Protein Data Bank (PlGF PDB ID: 1RV6, VEGF PDB ID: 1FLT) [44, 45]. Structures of nucleobases were obtained from the ZINC database, and the aptamer structures were developed using the macromolecule building tool in Schrodinger^®^. All structures were optimized using the protein preparation tool in Schrodinger prior to docking, which included preprocessing, H-bond optimization and minimization.

### Molecular docking

Nucleotides, dimers, and trimers were docked on the receptors using Schrodinger Glide standard precision docking. For PlGF, the grid was centered around GLU72 in chain V and SER58 in chain W, based on preliminary docking results. For VEGF, HIS86 in chain W was used as the grid center. The grid size was maintained at 36 Å for both receptors. The strongest binding nucleotides and short aptamers were screened based on the docking score, as more negative docking scores indicate stronger binding. The average docking score for the three strongest binding poses was used for all molecules.

Docking of long aptamers on the receptors was done using the Schrodinger protein-protein docking tool, which can be used to dock molecules with more than 300 atoms. Since this technique does not provide docking scores, long aptamers were also docked using Autodock vina [46]. The receptor grid size was set to 25 Å, and the docking site was selected based on short aptamer docking results. The binding strength of the conformations generated by protein-protein docking was evaluated using prime MM-GBSA energy. Prime MM-GBSA binding free energy is calculated using Eq. 1.

1 $$\eqalign{& \Delta {\rm{G}}\,{\rm{(binding)}}\,{\rm{ = }}\,{{\rm{E}}_{{\rm{complex,}}\,{\rm{minimized}}}}\, \cr & - \,({{\rm{E}}_{{\rm{receptor,}}\,{\rm{minimized}}}}\, - \,{{\rm{E}}_{{\rm{ligand,}}\,{\rm{minimized}}}}) \cr}$$

### Molecular dynamics simulations

Molecular dynamics (MD) simulations were performed for selected aptamers bound to the receptors using Schrodinger Desmond. Protein-aptamer complexes, which were generated by Schrodinger protein-protein docking, were prepared and solvated using the system builder tool. The solvation process employed the SPC solvent model and OPLS_2005 force field, and the system was neutralized by addition of Na^+^ or Cl^−^ ions. Molecular dynamics simulations were conducted for 100 ns with a recording interval of 100 ps, using an NPT ensemble at 300 K and 1.01325 bar. Prior to the simulation, the system was relaxed following the default relaxation protocol in Desmond. Upon completion of the simulation, Root Mean Square Deviation (RMSD) and Root Mean Square Fluctuation (RMSF) were analyzed using the Simulation Event Analysis tool in Desmond.

### Bio-layer interferometry

The binding kinetics of the aptamers on PlGF and VEGF were evaluated using the Sartorius Octet^®^ R4 system manufactured by Sartorius, USA. Anti-penta HIS (HIS1K) biosensors purchased from Sartorius, USA, were used for measurements. Aptamer solutions were prepared using 1X Sartorius Kinetics Buffer purchased from Sartorius, USA. Prior to the experiment, the biosensors were hydrated in a pH 7.4 Phosphate buffer purchased from VWR, USA, for 10 min. An initial baseline step of 60 s was done in pH 7.4 phosphate buffer since the protein stock solutions were dissolved in that buffer, and the proteins were loaded on the biosensors by immersing the biosensors for 300 s in protein solutions. PlGF and VEGF loading concentrations were kept at 2.5 µg/mL and 10 µg/mL respectively, since a significant binding response could be observed at those concentrations. It was followed by a secondary baseline step of 60 s and association and dissociation steps for 300 s each. The association and dissociation profiles of the aptamers were determined at concentrations of 0, 2.5, 5, 10, and 20 µM. A set of non-loaded reference sensors was used to mitigate the impact of non-specific binding. Binding parameters were calculated by Octet ^®^ Analysis Studio software using a 1:1 global-fitting model. All experiments were carried out in triplicate.

### Statistical analysis

Statistical analysis was conducted using JMP Pro 17 software. The statistical significance of the differences between Glide scores, MM-GBSA energies, and BLI parameters of aptamers was evaluated using Student’s t-test. Since the variances were unequal for BLI parameters, log_10_ values of the BLI parameters were used for the t-test.

## Conclusions

Preeclampsia remains a significant health concern for pregnant women and babies worldwide, with delayed diagnosis often leading to adverse outcomes for both the mother and the fetus. This study underscores the importance of the shift from traditional symptom-based diagnostics methods toward molecular diagnostics strategies. Using a combination of in-silico and in-vitro techniques, we designed and evaluated a set of aptamers with a high affinity towards VEGF and PLGF. The structural configurations of PlGF and VEGF proteins were acquired from the RCSB Protein Data Bank, while the aptamer structures were meticulously constructed and optimized using Schrodinger^®^. Through molecular docking, we discerned the robust binding characteristics of specific nucleotides to the proteins. Subsequent investigations on dimer and trimer combinations of these nucleotides revealed that trimers typically exhibited stronger binding than dimers. Our aptamer designs were refined to six distinct aptamer sequences, three for each protein, and the binding kinetics of these aptamers to his-tagged PlGF and VEGF proteins were then evaluated using the Sartorius Octet^®^ R4 system, shedding light on the interactions between the aptamers and proteins suggesting they can be used as effective sensing elements for Preeclampsia detection. By spotlighting VEGF and PlGF as potential biomarkers and harnessing the power of aptamers for their detection, we pave the way for more accurate, early, and efficient diagnostics solutions. Furthermore, our exploration into the binding dynamics of these aptamers offers a promising foundation for the development of critical aptamer-based point-of-care technologies for early preeclampsia detection. However, it is essential to acknowledge the limitations of the current study, including the need for further optimization of experimental conditions. In addition, the feasibility of using these aptamers for clinical sensing applications has not been evaluated yet and will be the focus of our next study. These steps will be crucial for validating the potential of these aptamers in clinical diagnostics.

## Electronic supplementary material

Below is the link to the electronic supplementary material.

Supplementary Material 1

## Data Availability

The data will be provided by the corresponding author upon reasonable request.
