# Supplementary material for: Systematic design and evaluation of aptamers for VEGF and PlGF biomarkers of Preeclampsia
Source: BMC Biotechnol. 2024 Sep 27;24:64. doi: 10.1186/s12896-024-00891-0 (PMC11428563; doi:10.1186/s12896-024-00891-0)
Supplement: Supplementary file 1 — Supplementary Material 1 [file 12896_2024_891_MOESM1_ESM.docx]

**Supplementary Information**

**Systematic Design and Evaluation of Aptamers for VEGF and PIGF Biomarkers of Preeclampsia**

Samavath Mallawarachchi^a,†^_,_ Rümeysa E. Cebecioglu^b,f,†^, Majed Althumayri^d,e,†^, Levent Beker^c^, Sandun Fernando^a^, Hatice Ceylan Koydemir*^d e^

*^a^* Department of Biological and Agricultural Engineering, Texas A&M University, College Station, TX 77843

*^b^* Department of Biomedical Sciences and Engineering, Koç University, Rumelifeneri Yolu, Sarıyer, Istanbul, 34450 Turkey

^c^ Department of Mechanical Engineering, Koç University, Rumelifeneri Yolu, Sarıyer, Istanbul, 34450 Turkey

^d^ Department of Biomedical Engineering, Texas A&M University, College Station, TX, 77843, USA

^e^ Center for Remote Health Technologies and Systems, Texas A&M Engineering Experiment Station, College Station, TX, 77843, USA

^f^Medical Laboratory Techniques, Health Services of Vocational School, Kent University, Istanbul, 34333, Turkey

*^†^Contributed equally*

a)
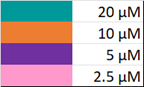

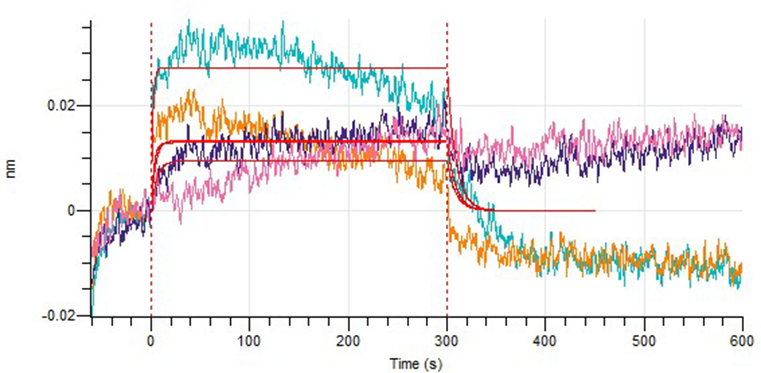


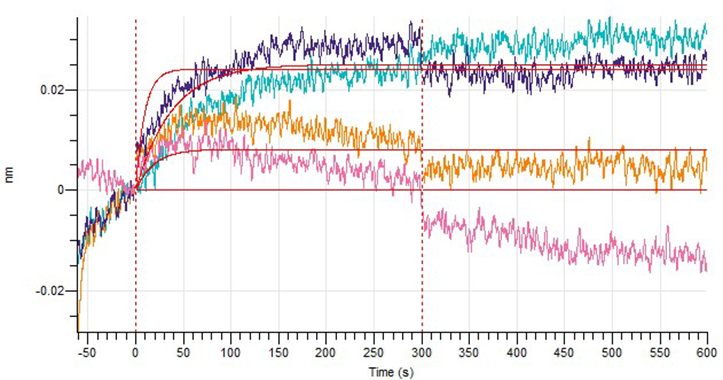


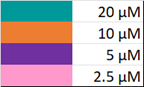


b)


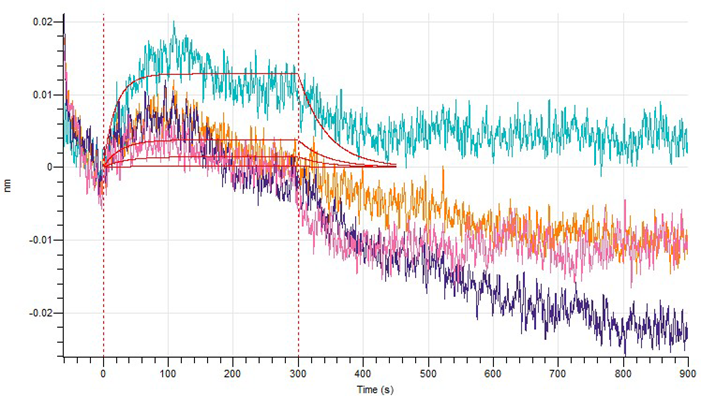


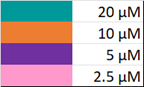


c)

**Fig. S1.** Association and dissociation curves for the binding of a) PlGF-Apt2, b) PlGF-Apt3, and c) PlGF-Apt4 on PlGF. Association occurs during the first 300 s, and dissociation occurs during the last 600 s.

1.
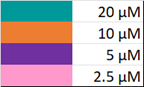

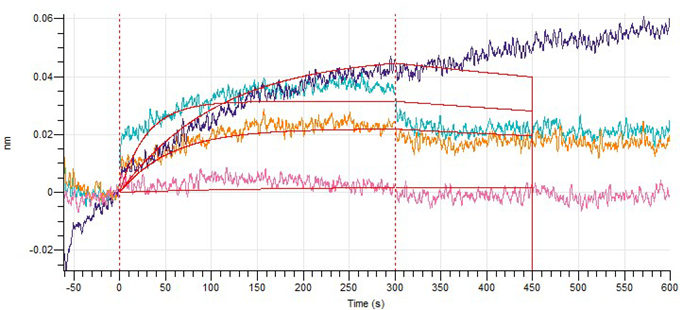

2.
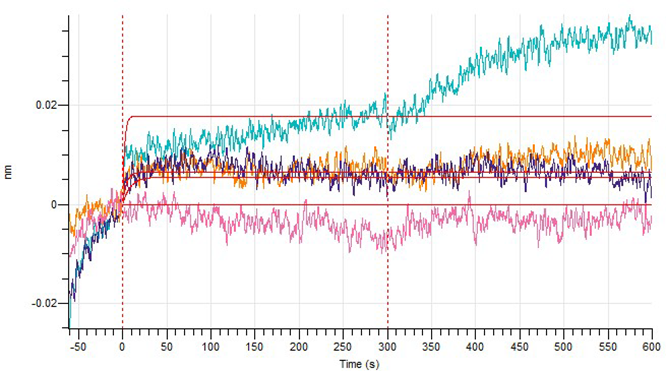


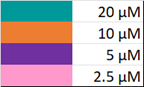


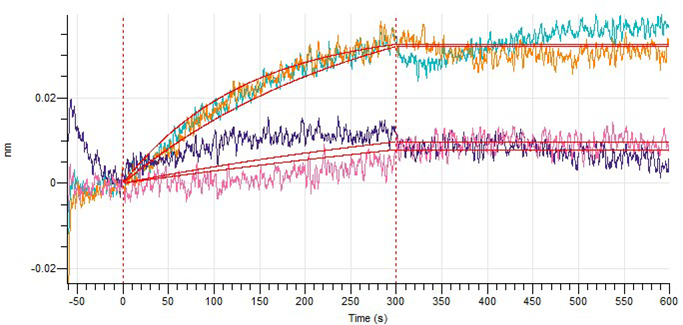


1.
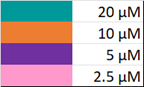


**Fig. S2.** Association and dissociation curves for the binding of a) VEGF-Apt1, b) VEGF-Apt2, and c) VEGF-Apt3 on VEGF. Association occurs during the first 300 s, and dissociation occurs during the last 600 s.

**Table S1.** Student’s t-test results for Glide docking energies of nucleotides on PlGF.

| Connecting Letters* Report | | | | | | |
| --- | --- | --- | --- | --- | --- | --- |
| Level |  |  | Mean | | | |
| UMP | A |  | -30.13033 | | | |
| GMP |  | B | -35.15300 | | | |
| CMP |  | B | -35.86233 | | | |
| AMP |  | B | -35.91133 | | | |
| Ordered Differences Report | | | | | | |
| Level | -Level | Difference | Std Err Diff | Lower CL | Upper CL | p-value |
| UMP | AMP | 5.781000 | 1.149620 | 3.12997 | 8.432029 | 0.0010 |
| UMP | CMP | 5.732000 | 1.149620 | 3.08097 | 8.383029 | 0.0011 |
| UMP | GMP | 5.022667 | 1.149620 | 2.37164 | 7.673695 | 0.0024 |
| GMP | AMP | 0.758333 | 1.149620 | -1.89270 | 3.409362 | 0.5280 |
| GMP | CMP | 0.709333 | 1.149620 | -1.94170 | 3.360362 | 0.5544 |
| CMP | AMP | 0.049000 | 1.149620 | -2.60203 | 2.700029 | 0.9670 |

*Letters not connected by the same letter are significantly different.

**Table S2.** Student’s t-test results for Glide docking energies of nucleotides on VEGF.

| Connecting Letters* Report | | | | | | |
| --- | --- | --- | --- | --- | --- | --- |
| Level |  |  | Mean | | | |
| UMP | A |  | -22.57267 | | | |
| CMP |  | B | -25.10833 | | | |
| GMP |  | B | -25.14500 | | | |
| AMP |  | B | -25.74333 | | | |
| Ordered Differences Report | | | | | | |
| Level | -Level | Difference | Std Err Diff | Lower CL | Upper CL | p-value |
| UMP | AMP | 3.170667 | 1.005968 | 1.30002 | 5.041312 | 0.0136* |
| UMP | GMP | 2.572333 | 1.005968 | 0.70169 | 4.442979 | 0.0338* |
| UMP | CMP | 2.535667 | 1.005968 | 0.66502 | 4.406312 | 0.0358* |
| CMP | AMP | 0.635000 | 1.005968 | -1.23565 | 2.505645 | 0.5455 |
| GMP | AMP | 0.598333 | 1.005968 | -1.27231 | 2.468979 | 0.5684 |
| CMP | GMP | 0.036667 | 1.005968 | -1.83398 | 1.907312 | 0.9718 |

*Letters not connected by the same letter are significantly different.

**Table S3.** Student’s t-test results for affinity constant (K_D_) of aptamers binding to PlGF.

| Connecting Letters* Report | | | | | | |
| --- | --- | --- | --- | --- | --- | --- |
| Level |  |  | Mean | | | |
| PLGF_Apt4 | A |  | -4.443488 | | | |
| PLGF_Apt2 | A |  | -4.904396 | | | |
| PLGF_Apt3 |  | B | -9.582594 | | | |
| Ordered Differences Report | | | | | | |
| Level | -Level | Difference | Std Err Diff | Lower CL | Upper CL | p-value |
| PLGF_Apt4 | PLGF_Apt3 | 5.139105 | 0.3717209 | 4.18357 | 6.094644 | <.0001* |
| PLGF_Apt2 | PLGF_Apt3 | 4.678198 | 0.3324773 | 3.82354 | 5.532858 | <.0001* |
| PLGF_Apt4 | PLGF_Apt2 | 0.460908 | 0.3717209 | -0.49463 | 1.416447 | 0.2700 |

*Letters not connected by the same letter are significantly different.

**Table S4.** Student’s t-test results for dissociation rate (K_d_) of aptamers binding to PlGF.

| Connecting Letters* Report | | | | | | |
| --- | --- | --- | --- | --- | --- | --- |
| Level |  |  | Mean | | | |
| PLGF_Apt2 | A |  | -0.714244 | | | |
| PLGF_Apt4 | A |  | -1.014339 | | | |
| PLGF_Apt3 |  | B | -6.276427 | | | |
| Ordered Differences Report | | | | | | |
| Level | -Level | Difference | Std Err Diff | Lower CL | Upper CL | p-value |
| PLGF_Apt2 | PLGF_Apt3 | 5.562183 | 0.3482159 | 4.66707 | 6.457300 | <.0001* |
| PLGF_Apt4 | PLGF_Apt3 | 5.262088 | 0.3893172 | 4.26132 | 6.262860 | <.0001* |
| PLGF_Apt2 | PLGF_Apt4 | 0.300095 | 0.3893172 | -0.70068 | 1.300867 | 0.4756 |

*Letters not connected by the same letter are significantly different.

**Table S5.** Student’s t-test results for affinity constant (K_D_) of aptamers binding to VEGF.

| Connecting Letters* Report | | | | | | |
| --- | --- | --- | --- | --- | --- | --- |
| Level |  |  | Mean | | | |
| VEGF_Apt1 | A |  | -5.121231 | | | |
| VEGF_Apt3 |  | B | -8.240837 | | | |
| VEGF_Apt2 |  | B | -9.827153 | | | |
| Ordered Differences Report | | | | | | |
| Level | -Level | Difference | Std Err Diff | Lower CL | Upper CL | p-value |
| VEGF_Apt1 | VEGF_Apt1 | 4.705922 | 0.9702794 | 2.33173 | 7.080110 | 0.0029* |
| VEGF_Apt1 | VEGF_Apt3 | 3.119606 | 0.9702794 | 0.74542 | 5.493794 | 0.0182* |
| VEGF_Apt3 | VEGF_Apt2 | 1.586316 | 0.9702794 | -0.78787 | 3.960504 | 0.1532 |

*Letters not connected by the same letter are significantly different.

**Table S6.** Student’s t-test results for Dissociation rate (K_d_) of aptamers binding to VEGF.

| Connecting Letters* Report | | | | | | |
| --- | --- | --- | --- | --- | --- | --- |
| Level |  |  | Mean | | | |
| VEGF_Apt1 | A |  | -2.583891 | | | |
| VEGF_Apt2 |  | B | -6.327780 | | | |
| VEGF_Apt2 |  | B | -6.369112 | | | |
| Ordered Differences Report | | | | | | |
| Level | -Level | Difference | Std Err Diff | Lower CL | Upper CL | p-value |
| VEGF_Apt1 | VEGF_Apt3 | 3.785221 | 0.3175308 | 3.00825 | 4.562191 | <.0001* |
| VEGF_Apt1 | VEGF_Apt2 | 3.743889 | 0.3175308 | 2.96692 | 4.520859 | <.0001* |
| VEGF_Apt2 | VEGF_Apt3 | 0.041332 | 0.3175308 | -0.73564 | 0.818302 | 0.9007 |

*Letters not connected by the same letter are significantly different.

**Table S7.** Comparison of aptamer sequences designed in this study to some of those reported in the literature.

| **Protein** | **Aptamer sequence** | **Affinity constant (KD)** | **The method used to estimate parameters** | **Target application** | **Reference** |
| --- | --- | --- | --- | --- | --- |
| VEGF | 5'-GCACTCTGTGGGGGTGGACGGGCCGGGT-3' | 20 nM | SPR  (using VEGF_165_) | Cancer diagnosis | [S1] |
| VEGF | 5'-GCACTCTGTGGGGGTGGACGGGCCGGGT-3' | 1.0 nM | SPR  (using VEGF_121_) | Cancer diagnosis | [S1] |
| VEGF | 5 -GGG CCG TTC GAA CAC GAG CAT GGT GGG TGG TGG CCC TAG GAT GAC CTG AGT ACT GTC C-3 | 315 nM | SELEX | Cancer diagnosis | [S2] |
| VEGF | 5'-TGTGGGGGTGGACGGGCCGGGTAGA-3' | 1.1 nM | SPR  (using VEGF_165_) | Cancer diagnosis | [S1] |
| VEGF | 5'-TGTGGGGGTGGACGGGCCGGGTAGA-3' | 1.4 nM | SPR  (using VEGF_121_) | Cancer diagnosis | [S1] |
| *VEGF* | *5'-GAAAGGCGA-3'* | *6.804 ± 4.655 nM* | *BLI*  *(using his-tagged protein)* | *Preeclampsia* | *This study* |
| *PlGF* | *5'-AGAGAACGCAAGAGA-3'* | *0.2983 ± 0.1815 nM* | *BLI*  *(using his-tagged protein)* | *Preeclampsia* | *This study* |

**Trajectory videos**

Trajectory videos are added to the Zenodo repository and can be accessed through this link.

<https://zenodo.org/records/13333661>

Supplementary References:

1. Nonaka Y., Sode K., Ikebukuro K., Screening and Improvement of an Anti-VEGF DNA Aptamer. *Molecules*.2010; 15, 215-225. doi:10.3390/molecules15010215
2. Qureshi A., Gurbuz Y., Niazi J. H. Capacitive aptamer–antibody based sandwich assay for the detection of VEGF cancer biomarker in serum. *Sensors and Actuators B: Chemical*. 2015; 645-651. https://doi.org/10.1016/j.snb.2014.12.040
